# The Diamond Disks

**Published:** 1877-04

**Authors:** 


### THE DIAMOND DISKS.

"How do you like them?" Very well, appreciate them more every day we use them. They do what no other disks will do. We have used one for about five months and it now cuts as well as at first. This disk is one of the good new things.

